# Human CD36: Gene Regulation, Protein Function, and Its Role in Atherosclerosis Pathogenesis

**DOI:** 10.3390/genes16060705

**Published:** 2025-06-13

**Authors:** Monika Rac

**Affiliations:** Department of Biochemistry, Pomeranian Medical University, Powstancow Wielkopolskich 72, 70-111 Szczecin, Poland; monika.rac@pum.edu.pl

**Keywords:** CD36, atherosclerosis, ox-LDL receptor

## Abstract

Human CD36 plays an important role in ligand binding, signalling, cell adhesion, and the regulation of angiogenesis. As a scavenging receptor, it is responsible for clearing long-chain fatty acids (LCFAs) and removing approximately 50% of oxidised low-density lipoprotein (ox-LDL) from plasma. The *CD36* gene is alternatively spliced. It has several alternative promoters and first exons. The alternative transcripts are expressed in multiple tissues, and their expression patterns are highly variable. The molecular mechanisms that regulate *CD36* gene expression are complex and reflect its multifunctional role in different tissues. CD36 activity has been linked to several metabolic processes, such as inflammation, angiogenesis, phagocytosis, and energy homeostasis. CD36 plays a key role in regulating vascular and cardiovascular health and in the pathogenesis of atherosclerosis. *CD36* gene mutations in the Caucasian population are rare. Hence, it is extremely difficult to recruit a statistically significant group of CAD patients with these mutations. Nevertheless, this population is largely at risk of cardiovascular disease. Atherosclerosis is a multifactorial disease, but the role of the CD36 receptor in the development of ox-LDL is extremely important. This review aims to introduce readers to issues related to the relationship between CD36 and CAD. The activity of this receptor should be considered when exploring treatment options for atherosclerosis-related complications.

## 1. Introduction

Atherosclerosis in the coronary arteries is associated with disturbances in lipid metabolism, inflammation, and the immune state in macrophages, endothelium, and smooth muscle cells. Disturbances in plasma lipid metabolism play a crucial role in macrophage activation and atherosclerotic plaque formation. Coronary atherosclerosis is a complex pathological process that eventually becomes life-threatening due to vascular abnormalities. During this long process, many pathophysiological factors influence the outcome of the disease. In the atherosclerotic plaque, many circulating immune cells with chemotaxis participate in endothelial injury and lipid infiltration. Coronary artery disease (CAD) is considered a clinical consequence of atherosclerosis, manifesting as a chronic inflammatory condition involving the release of platelet mediators [1]. The literature used to prepare this review is available in the PubMed database. The database was searched using the keywords of the individual chapters of this manuscript. Publications that were not related to human CD36 were excluded. Where possible, the search was limited to the last five years.

### 1.1. The Formation of Atherosclerotic Changes in Vessels

The endothelium allows atherogenic lipoproteins to enter the subendothelial space, where they initiate the formation of atherosclerotic plaques and undergo storage and modification. There, they are stored and modified. This modification, for example, in the form of lipoprotein oxidation, makes them more atherogenic. Oxidised LDL particles (oxLDL) have cytotoxic effects by binding to receptors on the macrophage membrane. This process leads to intracellular lipid accumulation and the formation of foam cells. In the early stages of atherosclerosis, the focal accumulation of monocytes, and to a lesser extent, T and B lymphocytes in the inner membrane of arteries, plays an important role. The migration of inflammatory cells begins with their adhesion to the endothelium [2]. Their transition to the middle membrane requires the activity of chemokines (chemotactic cytokines), including monocyte chemoattractant protein (MCP-1). Activated monocytes present in the middle membrane can secrete several cytokines that strongly influence the local accumulation and function of pro-inflammatory cells. Increased expression of the chemoattractant genes M-CSF (macrophage colony-stimulating factor) and MCP-1 also intensifies monocyte migration. In the vessel wall, monocytes are transformed into macrophages, which accumulate oxLDL and become overloaded with fats [3]. Inflammatory mediators released by macrophages—interleukin 1b (IL-1b), tumour necrosis factor (TNF), and M-CSF—intensify the binding of LDL to the endothelium and vascular muscle. Macrophages can initiate the oxidation of LDL particles and absorb oxLDL particles via scavenger receptors. Atherosclerosis progression is accompanied by a fibroproliferative reaction. Vascular smooth muscle cells play an important role in this reaction. There is also an accumulation of lipids in the extracellular space. This results from the retention of atherogenic lipoproteins in the extracellular matrix, as well as from the necrosis and apoptosis of foam cells [4]. Intracellular lipids are released into the extracellular space. This step involves the lipid-rich core of the atherosclerotic plaque. This space contains macrophages and foam cells with cholesterol deposits. This core is covered by a fibrous cap containing smooth muscle cells. The progression of the disease is promoted by neovascularisation and the expression of leukocyte adhesion molecules, such as VCAM-1 (vascular cell adhesion molecule 1) and ICAM-1 (intercellular adhesion molecule 1) [5]. The development of atherosclerosis is therefore a continuously reinforced process, and its essential substrate and regulator are oxLDL particles. Some atherosclerotic plaques pose a particular risk. These are unstable plaques. They are associated with a high risk of thrombosis in the vessel lumen. The most common cause of acute coronary syndromes, such as myocardial infarction, unstable angina, and sudden cardiac death, is the rupture of unstable plaques, along with the thrombotic process. The three main factors contributing to plaque rupture are lipid core size, inflammation, and impaired healing [6].

### 1.2. The Function of Scavenger Receptors, Including CD36

The uptake of modified LDL particles by macrophages involves various receptors, including scavenger receptors, which are located on mononuclear phagocytic cells. Scavenger receptors are a diverse group of proteins that are primarily expressed in macrophages and dendritic cells. These receptors are proteins that bind to chemically or oxidatively modified lipoproteins, polyanions, and apoptotic cells. There are six classes of SR (A to F). SR-AI and SR-AII bind to acetylated and oxidised LDL, as well as polyanions and dead cells [7]. Class B receptors include CD36 and BI receptors. SR-BI is a receptor for high-density lipoprotein (HDL). The SR-CI receptor binds to acetylated LDL and polyanions. Class D receptors capture oxLDL. Class E receptors (SR-LOX-1) bind to oxLDL and polyanions, while class F receptors capture oxLDL, acetylated LDL, and polyanions. Scavenger receptors play an important role in many physiological and pathological processes. Previous studies have found that some SR receptors are specifically involved in the formation of foam cells. These include the following receptors: SR-A, CD36, SR-D, LOX-1, SREC, and SR-PSOX [8,9,10]. This review aims to introduce readers to issues related to the relationship between CD36 and CAD.

Several proteins are involved in the cellular uptake of long-chain fatty acids (LCFAs), including plasma membrane fatty acid binding protein (FABP) and another protein called fatty acid translocase (FAT), also known and referred to as scavenger receptor cluster of differentiation 36 (CD36), leukocyte differentiation antigen CD36, glycoprotein IIIb (GPIIIB), platelet glycoprotein IV (GPIV), PAS-IV, PAS-4, platelet collagen receptor, or at least thrombospondin receptor. Receptor CD36 is a membrane glycoprotein found on platelets, monocytes, macrophages, endothelial cells, neurones, mammary epithelial cells, adipocytes, liver, kidney, haematopoietic cells, monocytes, and cardiomyocytes. It plays an important role in ligand binding, signal transduction, cell adhesion, and the regulation of angiogenesis. CD36 is a scavenger receptor that recognises and transports oxidised lipoproteins such as ox-LDL and fatty acids, but is also a receptor for thrombospondin-1 and *Plasmodium falciparum* [11,12,13].

## 2. Human *CD36* Gene

### 2.1. Structure of CD36 Gene

The human *CD36* gene is highly polymorphic. According to the NCBI database https://www.ncbi.nlm.nih.gov/gene/948/#gene-expression (accessed on 30 April 2025) [14], the human *CD36* gene has ID 948, is located on chromosome 7 q11.2, and is encoded by 15 exons. The *CD36* gene is expressed as 38 different transcripts, has different tissue locations, and varies in length and number of nucleotides. Interestingly, exons 1–2 and 15 are non-coding. The 5′ untranslated region of the CD36 mRNA is encoded by three exons up to 89 nucleotides. The next fragment encodes the N-terminal cytoplasmic and transmembrane domains. The next exons encode the extracellular domain. Exon 14 encodes the C-terminal domains of the CD36 protein, but the 3′-untranslated region is present only in exon 14 or in exons 14 and 15. The structure of the *CD36* gene was first described by Armesilla et al. [15]. The gene structure with the classification of exons and introns in the *CD36* gene, with corresponding amino acids (AA), is shown in Table 1.

### 2.2. CD36 Gene Alternative Splicing

One of the most distinctive features of the *CD36* gene is the presence of alternative and independent first exons and their promoters. According to Table 1, the first mRNA nucleotide encoding the CD36 protein is +1. At position 1709 within exon 14, there is an internal splice donor site that can bind nucleotide 1419 to the first nucleotide of exon 15, thus generating the alternative *CD36* mRNA form containing exon 15. Alternative splicing of the first *CD36* exon is regulated differently in different tissues, suggesting that the promoters are tissue-specific (platelets, monocytes, macrophages, endothelial cells, adipocytes, dendritic cells, muscle cells, liver, and haematopoietic cells). The alternative transcripts are expressed in more than one tissue, and their expression patterns are highly variable, which accounts for some of the heterogeneity in the molecular size of *CD36* mRNA and why the *CD36* gene is expressed as 38 different transcripts [16].

### 2.3. Regulation of CD36 Gene Expression

The functional diversity of CD36 results from alternative splicing of the *CD36* pre-mRNA. The molecular mechanisms regulating *CD36* gene expression are complex and reflect the multifunctional role of CD36 in different tissues, stimuli, and conditions [17]. In monocytes, CD36 receptor expression is upregulated by native and modified LDL, such as oxLDL, as well as adhesion and action of cytokines, cellular cholesterol, insulin, glucose, and interleukin-4 [18]. In adipocytes, the key feedback regulator of the *CD36* gene is the nuclear peroxisome proliferation activator receptor γ (PPARγ) [19]. The presence of PPARγ is necessary for the basic regulation of CD36 receptor expression. PPARγ is a regulator of gene transcription that encodes proteins involved in adipogenesis and lipid metabolism. Phosphorylation, through which many substances act, alters the transcriptional activity of PPARγ. These substances include growth factors such as EGF (epidermal growth factor) and PDGF (platelet-derived growth factor), which cause the phosphorylation of serine 82 on PPARγ via MAPK (mitogen-activated protein kinase). Phosphorylation significantly inhibits the transcriptional activity of PPARγ [20]. PPARγ ligands, such as the prostaglandin D_2_ metabolite 15-deoxy-D(12,14)prostaglandin, increase CD36 receptor expression. Two oxidised metabolites of linolenic acid that are responsible for activating PPARγ are: 9-hydroxyoctadecadienoic acid (9-HODE) and 13-hydroxyoctadecadienoic acid (13-HODE), which are components of the oxLDL molecule. Other activators of CD36 expression include GM-CSF (granulocyte-macrophage colony-stimulating factor), M-CSF, and IL-4 (interleukin-4). These factors also intensify expression at the PPARγ level. IL-4 acts through both PPARγ and by intensifying the action of 12/15 lipoxygenase. Induction of the latter results in increased production of 13-HODE and 15-HETE (15-hydroxyeicosatetraenoic acid), which are, in turn, transcriptional activators of PPARγ [21]. *CD36* expression in skeletal and cardiac muscle is increased by plasma fatty acids and triacylglycerides and by tissue energy demands. Expression is inhibited by corticosteroids, TGF-β, HDL, and lipopolysaccharides [22]. TGF-β reduces CD36 receptor expression through MAPK phosphorylation and subsequent PPARγ phosphorylation [23]. Statins reduce the concentration of PPARγ mRNA but also increase the activity of p44/42 MAPK, which catalyses PPARγ phosphorylation [24]. *CD36* expression is epigenetically regulated by DNA methylation and oxLDL-induced histone trimethylation in promoter regions. Furthermore, exposure of human monocytes to oxLDL induces epigenetic histone modifications that result in increased production of proinflammatory cytokines and chemokines (e.g., interleukins, TNF, and metalloproteins), leading to augmented foam cell formation [25].

There are two types of CD36 deficiency. Type I is characterised by a complete CD36 deficiency in all cell types, including platelets and monocytes, whereas type II is characterised by a CD36 deficiency in only platelets [26]. The frequency of CD36 deficiency varies widely between ethnic groups. It is more frequently observed in the Japanese, African, and Thai populations than in the European populations. The incidence of type I CD36 deficiency is 0.5–1%, whereas type II CD36 deficiency is approximately 3–11% in Japanese people, 8% in African Americans, and less than 0.4% in white Europeans. The Japanese Red Cross Blood Services even has a registry of CD36-negative blood donors with both types of deficiency [27,28]. Type I is extremely rare in Caucasians [29]. Researchers noted that type I deficiency results from missense and nonsense mutations located within the coding region or from mutations leading to increased CD36 mRNA instability, which would be predicted to give rise to the type I phenotype. Type II deficiency results from a post-transcriptional defect, such as aberrant tissue-specific *CD36* pre-mRNA splicing, splicing in the 5′ untranslated region, and the creation of a translational block [30]. It has also been suggested that the molecular mechanisms of type II CD36 deficiency are highly complex, with the possibility of additional genetic regulatory mechanisms. Yanai et al. [31] explained the convoluted phenotype-genotype correlation in type II CD36 deficiency by saying that an altered DNA sequence in the 3′-untranslated region or near the 3′-untranslated region may alter the structure of the mRNA and its stability in this type of CD36 deficiency. This specific allele may be located away from the *CD36* gene, as a cis or even trans element. General proteolysis of CD36 could account for either type I or type II deficiencies in platelets, but platelets are known to be remarkably resistant to proteolysis in healthy individuals. Platelet CD36 protein expression levels are regulated by other heritable platelet-restricting factors [32].

### 2.4. CD36 Gene Mutations

Based on our previous publication, we described a very precise, step-by-step sequence of cDNA *CD36* nucleotides with found and described mutations and AA sequences of the CD36 protein [33]. Type I CD36 deficiency is most commonly associated with the following homozygous or heterozygous mutations: *C268T*, *949insA,* and *329-330delAC*. The first is the most common mutation in Asians and accounts for more than 50% of the mutant allele frequency [34]. Individuals with type I CD36 receptor deficiency are at risk of developing anti-CD36 isoantibodies following transfusion or during pregnancy. Type II CD36 deficiency is probably heterogeneous and has been observed in individuals who do not carry any other CD36 mutation except for a heterozygous mutation (most commonly *C268T*). Table 2 shows the main CD36 gene mutations that cause the CD36-deficient phenotypes seen in Japan. Some authors suggest that polymorphisms in the *CD36* gene modulate lipid metabolism and cardiovascular risk in Caucasians [35,36]. In a population of nondiabetic individuals of Caucasian ancestry, Ma et al. [36] found that the haplotype represented by five polymorphisms: *−33137A-G, −31118A-G* (rs1761667), *25444G-A, 27645 del/ins*, and *30294C-G* (rs1049673) is associated with increased risk of coronary artery disease. Our team has been researching this issue for several years, and the results of some of our studies are presented below.

### 2.5. Metabolic Consequences of CD36 Mutations

The CD36 receptor is responsible for the removal of approximately 50% of ox-LDL from plasma. Ox-LDL plays a role in the transformation of macrophages into foam cells, but the absence of macrophage CD36 expression can result in the retention of ox-LDL in the plasma and the production of AGEs [41]. Long-chain fatty acids (LCFAs) are also the major ligand for the receptor. LCFAs are the main energy substrate for the heart, and their oxidation is important to ensure maximum cardiac performance, as the CD36 receptor acts as a major myocardial LCFA transporter [42]. LCFAs deficiency is a possible aetiology of hereditary hypertrophic cardiomyopathy (HCM), and the abnormal myocardial LCFAs metabolism seen in HCM patients may be related to abnormal CD36 molecules. It is not clear that reduced expression of *CD36* is vasculoprotective or that *CD36* gene mutations are associated with increased serum concentrations of total cholesterol and LDL. *CD36* expression in tissues with very active fatty acid metabolism (skeletal muscle, heart, mammary epithelium, and adipose tissue) and its involvement in foam cell formation (macrophages) suggest that lipoprotein binding to CD36 may contribute to the regulation of lipid metabolism with carnitine palmitoyltransferase I and the pathogenesis of atherosclerosis [43,44,45]. Tsubokawa and Kashiwagi et al. [46] showed a marked reduction in the uptake of ox-LDL by CD36-deficient macrophages, a finding suggesting that differences in atherosclerosis may occur in type I or type II CD36-deficient and CD36-positive individuals. Other authors [47] have observed abnormalities of lipids (increased plasma triglycerides or decreased HDL cholesterol) and glucose (impaired glucose tolerance or delayed response of insulin secretion and metabolism in type I CD36 deficiency). Homozygous or compound heterozygous mutations of the *CD36* gene in humans result in severe defects in cardiac uptake of LCFAs. Therefore, type I CD36 deficiency is closely associated with the absence of LCFAs accumulation and metabolism in the myocardium [48].

## 3. Human CD36 Protein

### 3.1. Structure of CD36 Protein

**Figure 1:**
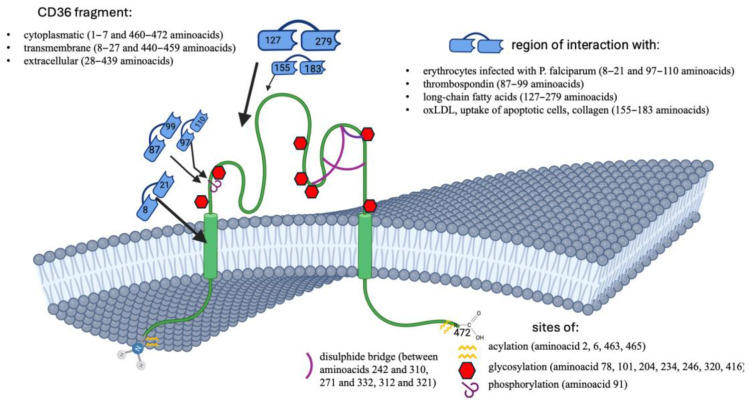
The structure of the CD36 protein.

According to the NCBI database [49], the CD36 protein has six different isoforms resulting from alternative splicing of transcripts of 317, 396, 412, 433, 438, or 472 amino acids in length. Protein glycosylation increases LCFAs uptake. The structure of CD36 is divided into five regions: carboxy-terminal (COOH-terminal) and amino-terminal cytoplasmic domains (NH2-terminal), two transmembrane regions, and an extracellular domain [50]. CD36 is located in the plasma membrane with a hairpin topology that spans the bilayer twice and ends in two short cytoplasmic tails. Post-translational modifications such as phosphorylation, palmitoylation, glycosylation, and ubiquitination of CD36 affect CD36 function and therefore LCFAs transport [51]. The extracellular domain is a large, highly glycosylated hydrophobic ring containing three pairs of disulfide bond sites. These modified sites can interact with a variety of extracellular substances, such as ox-LDL and LCFAs. Palmitic acid upregulates CD36 and promotes its translocation from the cytoplasm to the plasma membrane [52]. An extracellular region (Gly30-Asn439) of the CD36 protein is anchored to the plasma membrane by the transmembrane N-terminal (Gly8-Val29) and C-terminal (Leu440-Ile461) end domains. An intracellular region of 17 amino acids (Gly2-Cys7 and Ser462-Lys472) is critical for CD36 maturation and signalling activity. Human CD36 has 10 potential extracellular glycosylation sites: Asn79, Asn102, Asn134, Asn163, Asn205, Asn220, Asn235, Asn247, Asn321 and Asn417. The acetylation sites are Lys52, Lys166, Lys231, and Lys403. CD36 has four intracellular palmitoylation sites: Cys3, Cys7, Cys464, and Cys466. Finally, Lys469 and Lys472 are critical ubiquitination sites that target the protein for degradation [53,54]. Amino acid Lys164, exposed on the surface of the protein, is located in the region for palmitic acid dissociation and plays an important role in fatty acid uptake. The altered interactions induced by mutagenesis of this lysine have severely affected the folding, stability, form, utility, and solubility of the CD36 protein. Furthermore, acetylation of Lys166 may affect the LCFAs uptake activity of CD36 [55]. Figure 1 shows the structure of the protein. It also shows the ligand binding sites and potential sites for post-translational modifications.

### 3.2. CD36 Function

CD36 is a multifunctional membrane protein and a scavenger receptor that recognises and transports oxidised lipoproteins and fatty acids [56]. CD36 deficiency is relatively common in some populations and is estimated to be 2–9%.CD36 modulates tissue LCFA uptake. *CD36* expression is highly upregulated by PPARγ and is likely to be low when there is reduced LCFA activation of PPARγ [57]. This internalisation involves a kinase cascade initiated by the CD36 partner kinase Lyn, which results in CD36 depalmitoylation and endocytosis [58]. Whether CD36-mediated uptake, which is an important step in adipose, muscle, and cardiac LCFA metabolism, operates by the same mechanism is unclear. VEGF and its endothelial lipid receptor regulate endothelial lipid uptake. VEGF-A destabilises the interendothelial junctions, increasing the permeability of the barrier for lipids. CD36 has been shown to influence VEGF activation of VEGFR2 [59]. A high-fat meal increases *CD36* gene and protein levels in skeletal muscle in lean and obese people. A high glycaemic index (HGI) meal decreases CD36 mRNA and protein levels. Short bouts of endurance exercise boost the expression of the *CD36* gene and protein levels. *CD36* gene activation is associated with increased synthesis and activation of proteins that transport fatty acids, fatty acid oxidation, and lipid hydrolysis and intermediates: Uncoupled protein 3 (UCP3) gene, FABP4, long-chain fatty acid transport proteins 1 and 4 (FATP 1, FATP 4), carnitine palmitoyl transferase 1 (CPT 1), β-hydroxyacyl-CoA dehydrogenase (β-HAD), muscle lipoprotein lipase (mLPL), synthesis and activation of fatty acid transport proteins, oxidation of fatty acids, peroxisome proliferator-activated receptor (PPAR), nuclear-encoded protein peroxisome proliferator-activated receptor γ coactivator (PGC) 1, pyruvate dehydrogenase kinase (PDK) 4, citrate synthase (CS), 5-AMP-activated protein kinase (AMPK), extracellular signalling receptor kinase (ERK), and protein kinase C (PKC) [60].

Receptor CD36 is a major platelet protein of 80 to 90 kDa expressed in 10,000 to 25,000 copies on the platelet surface. CD36 is a signalling receptor and also acts as a transporter for long-chain fatty acids, thrombospondin, and oxLDL. The signalling activity of CD36 in platelets is a sensor for oxLDL in the circulation, which lowers the threshold for platelet activation in conditions of dyslipidaemia. Unfortunately, platelet CD36 translates atherogenic lipid stress into an increased risk of thrombosis, myocardial infarction, and stroke. CD36 inhibits the cyclic nucleotide signalling pathway while inducing paracrine platelet activation. This receptor also serves as a binding hub for several coagulation factors and contributes to the plasmatic coagulation cascade [61,62]. The scavenger receptor CD36, mainly found on macrophages, has a strong ability to take up oxLDL because it is not recognised by the LDL receptor. Excessive uptake of oxLDL by macrophages leads to increased cytokine secretion and activation of macrophages, which then become foam cells loaded with lipids, leading to plaque formation. When macrophages are overwhelmed by oxLDL, there is an imbalance between uptake and clearance of lipids, leading to increased inflammation, cellular necrosis, thinning of the fibrotic plaque, and ultimately plaque rupture and thrombosis [63,64]. In conclusion, in macrophages and other cell types, CD36 acts as a scavenger receptor for oxLDL and oxidised phospholipids. Such a role for CD36 has also been reported in platelets, along with other scavenger receptors such as SR-A [65,66].

CD36 plays a protective role in the body based on its function as a signalling receptor and fatty acid transporter. CD36 is also involved in various processes, including innate immunity, the removal of dead cells, and the uptake of infected cells [67].

### 3.3. CD36 Signalling and Regulation of the Paracellular Pathway

**Figure 2:**
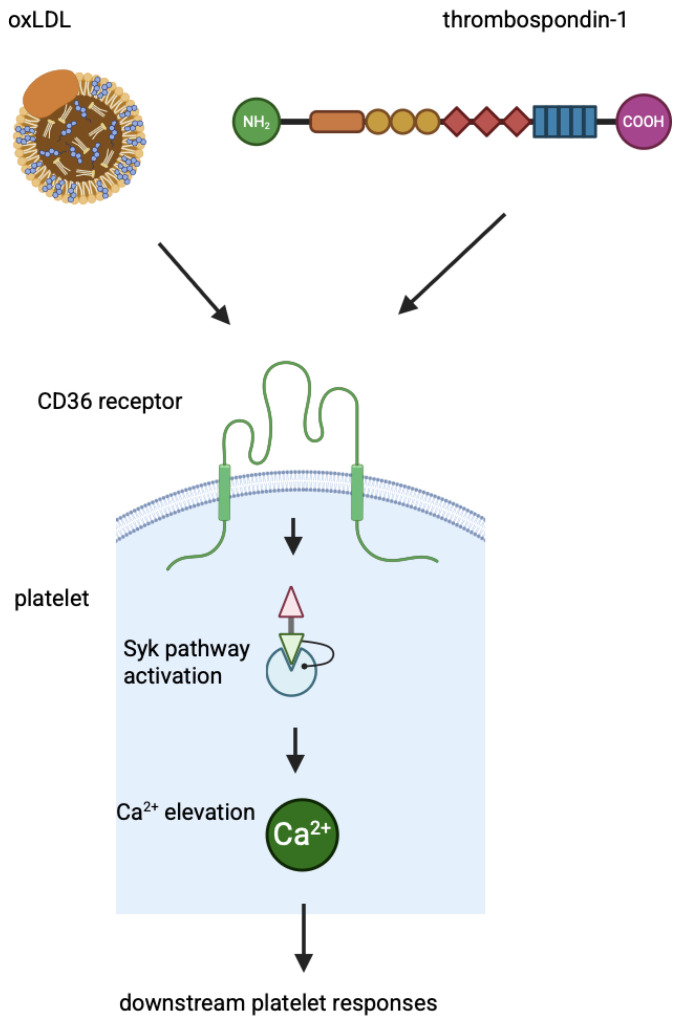
Signalling through CD36 in platelets.

Neither of the two CD36 domains has intrinsic phosphatase or kinase activity, binding sites for GTPases to transduce signals, or scaffolding domains. Therefore, to transduce signals, CD36 must initiate the assembly of a signalosome complex that includes intracellular and membrane protein partners. It is not clear how CD36 responds to different signalling partners when cells are exposed to different stimuli. It is possible that different ligands induce a variety of CD36 conformational changes and different effects. For example, cardiotonic steroids bind to the subunit of the Na/K-ATPase α1/Src signalling axis, inducing its interaction with CD36/TLR4 receptors and activating the NF-κB pathway, leading to pro-inflammatory responses in macrophages [68]. Fatty acids binding to CD36 activate several signalling effectors that control LCFAs metabolism: PPAR, cAMP/CAMKII/Ca^2+^, PLC/IP3/Ca^2+^, ERK1/2, PI3K/FOXO1, VEGFR2/AKT, IR/PI3K, and LKB1/AMPK signalling pathways [69]. The increased expression of CD36 in oxLDL-treated cells is mediated by the PPARγ, PKB/Akt, and NF-E2-related factor (Nrf2) signalling pathways. After internalisation by target cells, oxLDL degradation products interfere with mitogen-activated protein kinases (MAPKs) and the PI3K/Akt pathway [70]. Platelet activation, secretion, and spreading are mediated by the binding of thrombospondin-1 or oxLDL via the receptor CD36. The signal transduction triggered by the binding of thrombospondin-1 to platelets is the CD36-dependent signalling to protein tyrosine kinases, in particular Syk, leading to Ca^2+^ elevation and other downstream platelet responses, such as enhancement of collagen-dependent thrombus formation. This pathway is enhanced by autocrine ADP-mediated activation and integrin activation [71]. A mechanism of signalling through CD36 in platelets is shown in Figure 2.

**Figure 3:**
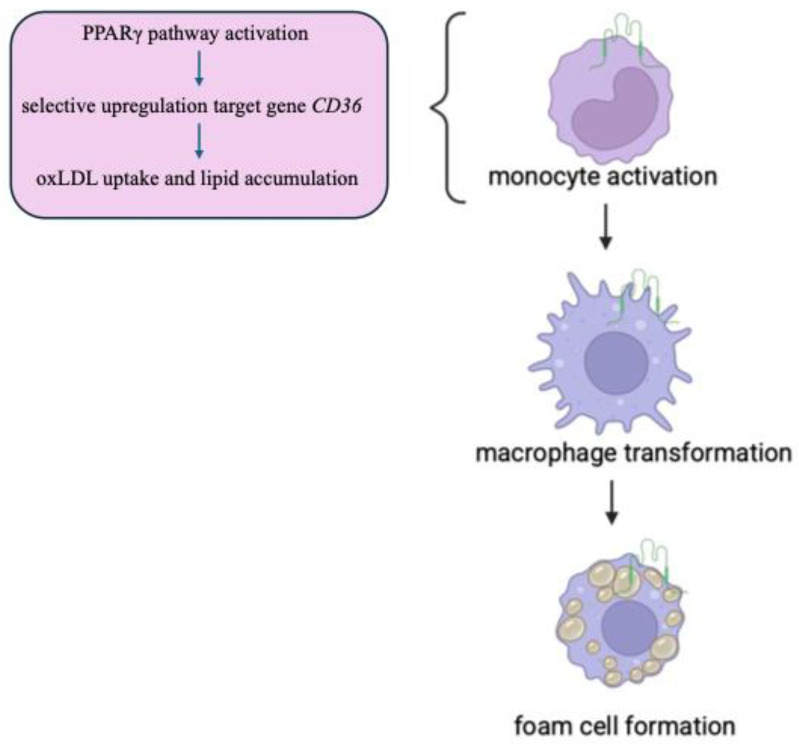
The signalling pathway triggered by oxLDL internalisation into the CD36 receptor.

PPARγ belongs to the nuclear receptor family of ligand-activated transcription factors and is most highly expressed in metabolic tissues, including white and brown adipose tissue. After binding to mono and polyunsaturated fatty acids, it is activated and heterodimerises with the nuclear receptor RXR to bind DNA and control transcription of target genes involved in lipid metabolism. LXRα gene expression can be upregulated by PPARγ ligands [72]. The regulatory mechanisms of the *CD36* gene transcription involve interactions with several transcription factors: peroxisome proliferator-activated receptor (PPAR) [73], CCAAT/enhancer-binding protein (C/EBP), and activating transcription factor 2 (ATF2). In addition, tissue-specific phosphorylation can modulate CD36 cellular location and ligand binding [74]. The target genes of the PPARγ pathway in macrophages are CD36 and FABP4, which are responsible for lipid uptake and accumulation in these cells. Thus, PPARγ selectively upregulates target genes that functionally result in increased lipid accumulation and oxLDL uptake in macrophages to facilitate foam cell formation [75,76]. Internalisation of oxLDL by CD36 leads to activation of PPARγ. PPARγ is highly responsive to oxidised fatty acid derivatives found in circulating oxLDL and has been implicated in macrophage cholesterol metabolism and inflammatory response. On this signalling axis, PPARγ upregulates target genes, such as fatty acid synthase and the scavenger receptor CD36 [73]. The signalling pathway triggered by oxLDL internalisation at the CD36 receptor is illustrated in Figure 3.

Other authors show that oxLDL binding to CD36 activates members of the MAPK family [77]. Moreover, various G-proteins mediate LCFAs to regulate mTORC1 signalling. Intracellular LCFAs translocated by CD36 are further metabolised to phosphatidic acid or acetyl-CoA, which regulates mTORC1 (mammalian target of rapamycin complex 1) activity. Whether mTORC1 senses intracellular LCFA levels or other existing LCFA sensors is unclear, but it is clear that CD36 is a receptor for LCFAs to mediate cellular LCFA signalling [78,79].

## 4. CD36 Function in Heart

### 4.1. Control of Energy Metabolite Access

In the healthy heart, CD36 and GLUT4 translocation to the cell surface is under the short-term control of the same physiological stimuli, such as increased contraction and insulin secretion. CD36 and GLUT4 move simultaneously from intracellular compartments to the cell surface. The fatty acids and glucose can be stored intracellularly in the heart, but most are immediately destined for mitochondrial oxidation and ATP generation to sustain contractile activity. There are control points in the metabolism of both substrates. For both substrates, cellular uptake is an important rate-controlling step in metabolism. In early-onset cardiac insulin resistance and chronic lipid overload, CD36 and GLUT4 behave in a bio-dysregulated manner. GLUT4 is sequestered intracellularly, and CD36 is shed to the cell surface. In palmitate-overloaded cardiomyocytes, changes in CD36 levels are visible within 1 h and precede changes in GLUT4 levels by 10 h [80,81,82]. Insulin resistance in heart muscle cells reduces their responsiveness to insulin, a critical hormone for glucose uptake and utilisation. This impairment of glucose metabolism leads to energy deficits and altered heart function, in particular, when combined with microvascular ischaemia, it increases the risk of cardiac hypertrophy, left ventricular dysfunction, and heart failure [83,84,85]. During the development of cardiac hypertrophy and failure, the heart switches to excessive glucose utilisation at the expense of fatty acids. A high-fat diet-induced increase in CD36-mediated fatty acid uptake rebalances myocardial fatty acids and glucose utilisation and restores cardiac contractile function [86]. Physical exercise increases the expression of PPARa in the heart, whereas cardiac pressure overload reduces it. The decrease in CD36 in pathological cardiac hypertrophy and the upregulation in physiological cardiac hypertrophy may be related to PPARa. It is known that insulin and contraction stimulation simultaneously promote the translocation of GLUT4 and CD36. Contraction stimulation is AMPK-dependent. The translocation of CD36 and GLUT4 helps to explain and distinguish the different energy regulation patterns of cardiomyocytes in the face of different signalling stimuli. In the cardiovascular system, CD36 is not only expressed on the surface of cardiomyocytes but is also present in other cells, such as endothelial cells. Therefore, a detailed study of the effects of CD36 on myocardial metabolism and in other cell types is an important issue [87].

### 4.2. LCFA Binding

CD36 takes up LCFAs in the cell membrane of cardiomyocytes for energy metabolism and is a master regulator of cardiovascular health. CD36 is also responsible for the transfer of fatty acids into the mitochondria. In normal physiology, the heart has a high capacity for fatty acid oxidation due to optimal CD36 activity. The peroxisome proliferator-activated receptor PPARγ induces *CD36* expression. Binding of CD36 receptor ligands activates downstream signalling pathways, fatty acid oxidation, and cellular calcium load. Overexpression of CD36 increases the fatty acid oxidation response by approximately 3-fold [88,89]. On the other hand, CD36 deficiency in the heart is compensated for by increased functional activity of lipoprotein lipase. As a result, CD36 deficiency in the human heart leads to increased circulating LCFAs levels, ketosis, and increased glucose metabolism. When fatty acid levels exceed the metabolic limit, CD36 becomes dysfunctional and is found in the plasma [90,91,92,93,94]. VLDL and chylomicrons are both lipoproteins that are rich in triglycerides (TG) of hepatic and dietary origin, respectively. In plasma, both lipoproteins release fatty acids and glycerol when exposed to lipoprotein lipase (LPL). LPL is located on the surface of the capillary endothelium. Albumin then transports the fatty acids in the plasma to the target cells. Studies also reveal that the uptake of lipids from VLDL and chylomicrons is not identical. Although LPL mediates lipid uptake from both TG-rich lipoproteins, the CD36 receptor in the heart only affects FA uptake from VLDL. CD36 has no effect on FA uptake from chylomicrons. One possible explanation for these differences is the much higher levels of non-esterified fatty acids released by chylomicrons. Consequently, the heart and brown adipose tissue require CD36 for the optimal uptake of VLDL-derived fatty acids [95,96,97]. Silencing CD36 expression or lipoprotein lipase reduces PPARα-mediated lipid deposition in cardiomyocytes. This leads to lipotoxic cardiomyopathy. The PPARγ pathway may be involved in the transport, storage, and release of FA in the heart, skeletal muscle, and adipose tissue. These tissues are exclusively dependent on PPARγ signalling [98,99]. In Dergunov et al.’s study [100], the increased expression of SREBF1 (sterol regulatory element binding transcription factor 1) is associated with a decrease in *CD36* gene expression. The profound decrease in *CD36* gene expression with an increase in HDL-C suggests that *CD36* gene expression contributes to the increased prevalence of CAD patients. This correlation has a sensitivity of 100% as a marker for CAD. Also, approximately 17% of Japanese patients with coronary heart disease and 40% of those with hypertrophic cardiomyopathy have CD36 deficiency [37].

### 4.3. CD36-Mediated Ca^2+^-Dependent Platelet Activation After Myocardial Infarction

The CD36 receptor regulates cytosolic Ca^2+^ and Ca^2+^-dependent platelet activation. After myocardial infarction, myocardial remodelling is influenced by overexpressed CD36-mediated Ca^2+^-dependent platelet activation. Store-operated Ca^2+^ channels are critical for maintaining myocardial health. CD36 knockdown delays cytosolic Ca^2+^ clearance, which is compensated by upregulation of SERCA2. Conduction abnormalities such as atrioventricular block and bradycardia are evident following CD36 knockdown, while tachyarrhythmogenic effects are caused by CD36 overexpression [101].

## 5. CD36 and Coronary Artery Disease (CAD)

My team examined a group of patients with early-stage coronary artery disease, young enough that the onset of CAD could be the result of genetic changes rather than correlating with age. We detected sequence changes in the *CD36* gene region encoding the oxLDL-binding and fatty acid domains in three exons 4–6 and their introns. The changes included six single nucleotide substitutions: in intron *3 IVS3-6 T/C* (rs3173798), in intron *4 IVS4-10 G/A* (rs3211892), in exon 5 *C311T*/Thr104Ile, in exon 6: *G550A*/Asp184Asn (rs138897347), *C572T*/Pro191Leu (rs143150225), *G573A*/Pro191Pro (rs5956), and *A591T*/Thr197Thr (rs141680676). To the best of our knowledge, we are the first to identify the polymorphism *C311T*. We did not report this mutation to the database, but we did receive an rs ID number, though. For all sequence changes, the genotype distributions were consistent with the Hardy–Weinberg equilibrium. The most common exonic sequence change is rs5956 and is similar to that described in the dbSNP base for Caucasian populations (4.2–4.5%). This polymorphism is located in a conserved splice site. The most common intron polymorphism, rs3173798, is also similar to that described in the dbSNP database for Caucasian populations (6.2–11.2%). This variant is much more common in Asian and African populations (21–41%) [102]. In our study [103], the frequency of *CD36* genotypes and haplotypes did not differ between patients with early-onset CAD and the no-CAD adult group, nor between neonates. Thus, the *CD36* analysed polymorphisms, rs3173798, rs3211892, rs138897347, rs5956, rs143150225, rs141680676, and *C311T*, do not seem to be involved in the risk of early-onset CAD in the Caucasian population. Population drift does not seem to be a problem in the study, as the frequency of *CD36* polymorphisms in newborns is similar to that in the group of over 70-year-old adults without CAD. On the other hand, some studies have shown that the C allele of rs3173798 tends to increase *CD36* expression, which correlates with a decrease in serum high-density lipoprotein levels and an increase in serum low-density lipoprotein levels [104]. In addition, a *C/T* substitution in exon 4 (rs75326924) in the *CD36* gene has been reported to be associated with a significant reduction in myocardial LCFAs uptake in patients with angina pectoris, myocardial infarction, hypertrophic cardiomyopathy, dilated cardiomyopathy, hypertension, aortic stenosis, and mitral valve disease, but only in the Japanese population [38].

In the CAD group, we demonstrated that the rs3173798 polymorphism of the *CD36* gene is associated with cardiovascular risk factors such as type 2 diabetes, high hsCRP, body mass index, and younger age at myocardial infarction [105]. The *IVS3-6TC* heterozygotes of this polymorphism had impaired left ventricular diastolic function (LVDF) more frequently than the wild-type homozygotes [106]. Polymorphism rs5956 was associated with lower plaque thickness and density in the common carotid artery. The ABI was lower in CAD patients with the rs141680676 polymorphism. When both variations were taken together as a haplotype, plaque thickness and density were lower [107]. The aim of the last study on the *CD36* gene [108] was to analyse the predictive values of the previously studied *CD36* gene polymorphisms over a 10-year follow-up period. It was the first published report with such a long-term follow-up of patients with CAD. There were no significant differences between the *CD36* variants and cardiovascular hospitalisation, myocardial infarction, all cardiovascular events, death during follow-up, cardiovascular death, and life expectancy. In conclusion, we have shown that the *CD36* variants we analysed do not appear to be associated with a risk of secondary cardiovascular events in patients with early CAD in the Caucasian population.

## 6. sCD36, Receptor Soluble in Plasma

There is also a circulating form of CD36 called soluble CD36 (sCD36). The mechanism of sCD36 generation is not entirely clear. Some have suggested that a plasma protease cleaves the extracellular segment of CD36, which includes sCD36. Other reports indicate that sCD36 is a full-length protein associated with a subset of small (0.1–1 µm in diameter) circulating microparticles. They are shed from the cell membranes of platelets, endothelial cells, erythrocytes, and leukocytes as a result of cell activation, senescence, and apoptosis [109]. Increased sCD36 levels were observed in pre-diabetes, e.g., impaired glucose tolerance. In non-diabetic patients, however, plasma sCD36 levels were strongly associated with fatty liver, carotid atherosclerosis, and insulin resistance [110,111]. The loss of weight leads to a decrease in the level of sCD36 [112]. In the analysis of patients with early CAD, there were negative correlations between sCD36 and ApoB/ApoA1 ratio, haemoglobin, red blood cell count, haematocrit and glucose concentration, as well as BMI, patient weight and waist circumference, WHR, MAP values and systolic blood pressure, as well as left ventricular end-diastolic diameter, left ventricular end-diastolic volume, left atrial diameter and right ventricular end-diastolic diameter, but positive correlations with ApoA1 and HDL cholesterol concentrations. The presented data suggest a possible protective effect of higher sCD36 concentration in relation to metabolic syndrome components, but not with the potential risk factor of impaired left ventricular diastolic function [113]. Strong associations between sCD36 and radiological parameters of atherosclerosis progression and risk of plaque instability were not observed [114]. In the study of children with hypercholesterolaemia, a protective effect of higher sCD36 levels was also observed in relation to components of the metabolic syndrome. There was a negative correlation of sCD36 with HOMA-IR ratio and insulin concentrations, plasma uric acid level, weight, BMI, waist and hip circumference, WHR, systolic blood pressure, and mean arterial pressure ratio, but a positive correlation with ApoA1 and HDL cholesterol concentrations. [115].

Although the current studies suggest that CD36 contributes to the development of T2DM on two levels, insulin resistance and pancreatic β-cell dysfunction and damage [116,117], the role of CD36 in the pathogenesis of DM is unclear. sCD36 is associated with T2DM risk factors such as metabolic syndrome. However, the heterogeneity of the populations studied and the lack of standardisation of sCD36 determination do not seem to support the use of sCD36 as a marker. There have been attempts to develop a reliable ELISA method for the determination of sCD36. This paper describes in detail each step of protein preparation and measurement, as well as the problems associated with this process [118]. In our other publication, we carefully described the function of CD36 in diabetes mellitus and its complications. The studies supported the role of CD36 in the pathogenesis of DM [119].

## 7. Perspectives in Diseases

Several events, such as inflammation, angiogenesis, phagocytosis, and energy homeostasis, have been linked to CD36 activity and its role in cellular senescence. Of relevance is the CD36-mediated induction of endoplasmic reticulum stress by oxLDLs in various cell types. Of note, oxLDL-induced cellular senescence can be counteracted by downregulation of CD36 expression, ROS scavenging, and inhibition of NADPH vitamin E [120,121,122]. It is therefore not surprising that research is also being carried out on other organs in the context of the function of CD36. Hyperlipidaemia worsens the outcome of ischaemic stroke by increasing *CD36* expression in the post-ischaemic brain and in peripheral macrophages. This infiltration contributes to stroke-induced brain damage [123]. The findings show that CD36 acts as an LCFA transporter and regulates LCFA oxidation, VLDL secretion, lipid synthesis, inflammation, and autophagy in liver cells. When CD36 increases LCFA uptake in the liver, it drives the onset of hepatosteatosis. In a cross-sectional clinical study in a healthy population, it was found that plasma sCD36 was also correlated with the presence of fatty liver [62,124]. The functional diversity and versatility of CD36 can mediate epithelial-mesenchymal transition in a wide range of tumour cells, promoting tumour progression and metastasis [125,126,127]. Cancer cells undergo metabolic reprogramming and switch from using glucose to using fatty acids for energy. The CD36 receptor is highly expressed in certain types of cancer cells. Its high expression in tumour cells triggers FA uptake and lipid accumulation, which promotes rapid tumour growth and initiates metastasis [128]. It is known that glutamine treatment increases FA uptake/oxidation and membrane CD36 levels [69]. Several CD36 blockers are currently available, including specific CD36 antibodies and certain naturally occurring products. Their efficacy needs to be validated in preclinical and clinical studies. Given the ubiquitous expression and cell-specific effects of CD36, the potential toxicity of targeting CD36 must be carefully considered to minimise potential side effects of any treatment [129,130,131,132].

In summary, CD36 is a multifunctional protein with key roles in lipid uptake, inflammation, immune regulation, angiogenesis, and tumor growth. PPAR-γ induces upregulation of the CD36 receptor and promotes macrophage differentiation and enhanced uptake of oxLDL. The differentiation of monocytes into macrophages is also modulated by cytokines such as IL-10 and IL-4. Increased binding of oxLDL to the CD36 receptor in macrophages is associated with atherosclerotic lesions. Blocking CD36 modulates the secretion of IL-1β, IL-6, and IL-8, as well as foam cell formation, in human macrophages [29]. Therefore, we can conclude that reducing the CD36 receptor prevents the development of atherosclerosis. This opens the scope for further research into the prevention of atherosclerosis in humans. However, a Japanese study examined 40 individuals deficient in the CD36 receptor. These individuals were found to have a significantly higher incidence of coronary heart disease than the general population, suggesting that CD36 deficiency may promote atherosclerosis [133]. The activity of this receptor should be considered when treatment options for atherosclerosis-related complications are being explored. Current CAD treatment does not consider the role of CD36. However, drugs that regulate CD36 activity may be developed in the future, in a similar way to PCSK9 inhibitors. In the vascular system, PCSK9 interacts with other receptors, including CD36. PCSK9 primarily causes the degradation of the low-density lipoprotein receptor (LDLR), thereby reducing cholesterol clearance. The binding of PCSK9 to the CD36 receptor activates Src kinase, MAPK, and C-Jun N-terminal kinase (JNK), thereby increasing reactive oxygen species (ROS) levels. This results in platelet activation and thrombosis [134,135]. In addition to regulating LDL-R, it appears that PCSK9 inhibitors can also bind to the scavenger receptor CD36 [136]. Authors reported that salvianolic acid B (SAB), a polyphenol compound extracted from the roots of Salvia miltiorrhiza, is a CD36 antagonist that blocks the uptake of oxidised lipids in macrophages, making CD36 a potential target for intervention in metabolic disorders. Inhibiting CD36 with SAB in mice with diet-induced obesity reduced visceral fat accumulation and improved insulin resistance [137]. Further research should concentrate on the treatment of patients with CAD. Moreover, both ox-LDL and high-fat diets, along with several long non-coding RNAs (lncRNAs) and miRNA, increased CD36 expression on aortic and coronary endothelial cells [138,139]. Epigenetic research and gene regulation by selected miRNAs are also worth focusing on.

## Figures and Tables

**Table 1:** The classification in terms of exons and introns in the *CD36* gene.

Exon Number	Next Intron Length	mRNA Nucleotides	Amino Acids Encoded
1	7341	−289 to −184	none
2	470	−183 to −90	none
3	9679	−89 to +120	1 to 40
4	4362	121 to 281	41 to 94
5	1779	282 to 429	95 to 143
6	1236	430 to 609	144 to 203
7	1945	610 to 701	204 to 234
8	3463	702 to 748	235 to 250
9	954	749 to 818	251 to 273
10	757	819 to 1006	274 to 336
11	729	1007 to 1125	337 to 375
12	511	1126 to 1199	376 to 400
13	573	1200 to 1254	401 to 418
14	2236	1255 to 1419	419 to 472
15	none	1420 to 2044	none

**Table 2:** The *CD36* gene mutations that are primarily responsible for causing CD36-deficient phenotypes in Japan, based on [27,35,37,38,39,40].

Exon	Rs Number	Nucleotide Change	Allele Frequency	Protein Change
int3/ex4	rs1165943635	121–126delgCAAGTT	not found	del AA 41–42 frameshift with the stop codon
4	rs75326924	C268T	3.5%	Missense P90→S
5	rs572295823	329–330delAC	1.2%	Frameshift at AA110
7	Chr7: 80,664,415–80,664,421	619–624delACTGCA/insAAAAC	1.8%	Frameshift at AA207 and a premature stop codon
9	Chr7: 80,669,953–80,670,022	749–818del 70 bp	not found	exon 9 skipping
9	rs142186404	T760C	not found	Missense F253→L
10	rs70961716	949insA	1%	Frameshift at AA317
int12/ex13	rs1261358979	1200_1202deltattacagAG	1%	Exon 13 skipping
13	rs550565800	1228_1239 delATT GTG CCT ATT	7.2%	Frameshift Mutation, 4 AA deletion
13	rs121918035	A1237C	not found	I413→L
